# Depression, anxiety and panic disorders in chronic obstructive pulmonary disease patients: correlations with tobacco use, disease severity and quality of life

**DOI:** 10.1186/s12971-017-0128-9

**Published:** 2017-04-07

**Authors:** Oana Irinel Pascal, Antigona Carmen Trofor, Lucia Maria Lotrean, Dumitru Filipeanu, Letitia Trofor

**Affiliations:** 1 “Grigore T. Popa” University of Medicine and Pharmacy Iasi, No 16 Universitatii Street, 700115 Iasi, Romania; 2 “Iuliu Hatieganu” University of Medicine and Pharmacy Cluj-Napoca, No 8 Victor Babeş Street, 400012 Cluj-Napoca, Romania; 3 “Gheorghe Asachi” Technical University Iasi, No 67 Profesor Dimitrie Mangeron Street, 700050 Iași, Romania

**Keywords:** Chronic obstructive pulmonary disease, Tobacco use, Depression, Anxiety, Panic disorders

## Abstract

**Background:**

The objective of this study is to assess anxiety, depression and panic disorders among patients diagnosed with COPD and to investigate their correlation with disease severity, quality of life as well as tobacco use.

**Methods:**

An observational study was performed between January and September 2014 among 60 patients diagnosed with COPD. COPD staging according to GOLD criteria, while anxiety and depression were assessed using Hospital Anxiety and Depression Scale and panic attacks were evaluated based on ICD 10 criteria.

**Results:**

Almost 40% of the sample were smokers, the medium packs-years was 34.3 and the medium Fagerstrom score was 7.5. Overall, mean Modified Medical Research Council Dyspnea Scale (mMRC) was 2.86, mean COPD Assessment Test (CAT) score was 21.75 and study participants had 1.93 COPD exacerbations/year. Mean distribution of anxiety and depression symptoms scores among COPD subjects was 10.65 ± 3.5 and 9.93 ± 3.8, respectively. Smokers and ex-smokers had similar scores with regard to anxiety, depression or the presence of panic attacks. The results of the bivariate correlations indicated associations between anxiety, depression, panic attacks and disease severity, as well as poor quality of life of patients with COPD, regardless of their current tobacco use status.

**Conclusions:**

In conclusion, the results of this study indicate that anxiety, depression and panic attacks were constant characteristics among COPD patients- regardless of their current tobacco use.

## Introduction

In chronic obstructive pulmonary disease (COPD), a systemic and disabling disorder, patients face a multitude of symptoms, together with a psychological load in relation with their disease [1, 2]. In recent years there is increasing evidence that COPD patients often experience panic attacks, anxiety and depression symptoms, especially in severe disease stages; when anxiety and depression occur, the risk of re-hospitalization and mortality is increased [1–4]. Moreover there is growing recognition of the fact that patients diagnosed with depression and bipolar disorders die prematurely due to concomitant illnesses [1, 2]. Explanation for this bilateral interaction lies in both symptom burden, functional impairment and psychological changes that accompany a chronic condition such as COPD for example, and in maladaptive health risk behaviors of the psychiatric patients [1, 2].

Symptoms of anxiety and depression are two of the most common co-morbidities in people with COPD [5], leading to significantly poor health outcomes, reduced quality of life and significantly increased healthcare costs [6]. In a retrospective, observational study, Gratziou and colleagues reported that the prevalence of depression among COPD patients with severe airway obstruction (FEV_1_ < 50%) was 25% and they had a 2.5 times greater risk of depression in comparison to healthy smokers [7]. High prevalence of depression is independently associated with smoking [8] and failure to quit [9]. Depression is also one of the withdrawal symptoms that predicts relapse to smoking [10]. Patients with anxiety and depression often suffer from low self-confidence or self-efficacy, which may lead to worsened disease related coping [11] and poor self-care behaviors. Moreover, co-existing addiction and psychiatric disorders significantly decrease the cessation success rates in COPD smokers and increase mortality among these patients [12].

Compared with persons without physical illness, COPD patients have impaired quality of life [13–15]. That is why numerous diagnostic tools, like the COPD Assessment Test (CAT) [16], St George’s Respiratory Questionnaire (SGRQ) [17], Cough and Sputum Assessment Questionnaire (CASA) [18] questionnaires, have been developed recently, to assess how daily life of the COPD patients is affected by their clinical condition.

In Romania, there are few data regarding the prevalence of COPD at national level. A study performed in 2012 estimated that 8.13% of population over 40 years had this condition [19]. Even less is known about co-morbid depression, anxiety and panic in Romanian COPD patients, except for a few data available [19]. Hence, the first objective of this study is to assess anxiety, depression and panic disorders among smoking patients diagnosed with COPD. Secondly, we will investigate their correlation with respiratory disease severity and quality of life as well as with tobacco use.

## Methods

### Study population

An observational study was conducted between January and September 2014, among patients diagnosed with COPD from the Clinic of Pulmonary Diseases from Iaşi, Romania. The study was approved by the local ethics and management boards, based on volunteer consent and permissions of participants and in respect to legislation for confidentiality of patient’s data use and publishing.

The inclusion criteria of the subjects were:

- aged > 40
- a valid diagnosis of COPD according to the ERS-ATS criteria for at least 12 months [20]
- had no history of psychiatric diagnosis other than depression, anxiety, or panic attack

### Tools and Measures

The subjects were asked to fill in a questionnaire which assessed demographic data, medical history (with special emphasis to cardiovascular co-morbidities), smoking status, COPD staging and issues related to anxiety, depression and panic attacks as well as the impact of COPD on their quality of life.

To define smoking, we used the standard Romanian Smoking Cessation Guideline brief questionnaire asking about past 12 months cigarettes consumption, number of packs-years (PY), previous quit attempts and including also nicotine dependence Fagerstrom test [21].

The 2011 “ABCD” GOLD guideline classification, was used to stage COPD. This classification was still in use in 2015 [20].

Anxiety and depression were screened by the Hospital Anxiety and Depression Scale (HAD scale), whilst the ICD 10 criteria served to confirm panic attacks. The HAD scale is a 14 item scale that generates ordinal data. Seven of the items relate to anxiety and seven to depression. Each item on the questionnaire is scored from 0 to 3 and this means that a person can score between 0 and 21 for either anxiety or depression, with scores categorized as follows: normal (0–7), mild (8–10), moderate (11–14) and severe (15–21) [22].

Impact of COPD on patients’ quality of life was determined by means of the COPD Assessment Test (CAT), a patient-completed instrument, designed to provide a simple and reliable measure of health status in COPD, to both physician and patient [16].

CAT is a simple 8 item questionnaire that measures the general impact of COPD on a patient’s health and how this is changing over time; it is recommended for use at any COPD visit. It contains questions about COPD symptoms-cough, phlegm, tight chest - exercise capacity and quality of sleep. It has a scoring range of 0-40 and implications of the score need to be evaluated in relation to a patient’s disease severity. Each patient gets a score at each visit. Any difference or change of 2 or more is considered meaningful for interpretation of changes in COPD status.

Dyspnea was evaluated through *Modified Medical Research Council Dyspnea Scale* (mMRC), a 0-4 gradually assessing dyspnea index.

#### Statistical Analysis

Descriptive analysis were performed, while Pearson bivariate correlations were used to assess the relationship between several variables. A statistically significant threshold was considered a *p* < 0. 05. Data were analyzed using SPSS 17 (SPSS Inc.).

## Results

A total of 60 COPD patients (52 men and 8 women) were enrolled in the study. Mean age was 62.2 years of age. A percentage of 38.3% were smokers and 61.7% were ex-smokers in the last 12 months). Among smokers, the medium packs-years was 34.3 and the medium Fagerstrom score was 7.5.

Cardiovascular co-morbidity (hypertension, coronary heart disease, acute myocardial infarction, heart failure) was identified in a large proportion (43.3%) of subjects.

In terms of COPD severity, as classified by the GOLD 2011 criteria, 23.3% were in stage B, 41.7% in stage C and 35% in stage D. The medium number of COPD exacerbations/year was 1.9 and the medium number of severe exacerbations was 0.8 per year. Overall, mean mMRC was 2.86 ± 0. 92 SD, mean CAT score was 21.75 ± 8.24 SD. Panic attacks, as judged by the ICD 10 criteria were recognized in 43.3% of patients. Anxiety and depression symptoms among COPD subjects were identified (10.65 ± 3.54 SD anxiety score, to 9.93 ± 3.80 SD depression score respectively). A percentage of 16.7% of participants had a normal score with regard to anxiety (score 0–7), 40% had mild anxiety (score 8–10), 30% moderate anxiety (score 11–14) and 13.3% severe anxiety (score 15–21). With regard to depression 23.3% had a normal (score 0–7), 31.7% had mild depression (score 8–10), 35% moderate anxiety (score 11–14) and 10% severe anxiety (score 15–21).

The results of the bivariate correlation analyses showed no significant correlations between smoking status (smokers vs ex-smokers) and either severity of COPD, depression, anxiety, or panic attack. Heavy smoking was not associated with COPD, depression, anxiety, or panic attack, in our sample, while cardiovascular co-morbidity history also did not influence HAD scale scores or any panic event as no significant correlation was distinguished.

On the other side, our results indicated that mMRC and CAT scores had a significant correlation (r = 0.63, p ≤ 0.001), while anxiety and depression (assessed by HAD scale) were found to correlate significantly (r = 0. 54, p ≤ 0.001). Moreover, mMRC scores also obtained significant correlations with the score for anxiety (r = 0.71, p ≤ 0.001), score for depression (r = 0.34, *p* = 0.019), and panic events (r = 0.551, p ≤ 0.001). Similarly, CAT’s scores had significant correlations with: the: score for anxiety (r = 0.63, p ≤ 0.001), score for depression (r = 0.45, *p* = 0.002), respectively with panic attacks (r = 0.386, *p* = 0.008). Finally, COPD Gold stages were correlated significantly with scores obtained for anxiety (r = 0.307, *p* = 0.001).

## Discussions

Our results indicated that anxiety and depression were constant findings among our COPD patients, however this was unrelated to tobacco use within this population.

In COPD, depression & anxiety prevalence estimates vary widely, due either to the use of varied measurement tools, or to the different degrees of illness severity across studies. In stable COPD, the prevalence of clinical depression ranges between 10 and 42%, while that of anxiety ranges between 10 and 19%. The risk of depression is higher in patients with severe COPD compared to control subjects and especially in oxygen-dependent patients, as well as in patients who have recently recovered from an acute COPD exacerbation [23]. In another systematic review of 64 studies focusing on patients with severe COPD, it was concluded that the prevalence of depression ranged from 37 to 71%, and that of anxiety from 50 to 75%, figures comparable to or higher than prevalence rates in other severe somatic conditions, such as cancer, AIDS, heart disease, and renal disease [24, 25].

Many patients with COPD experience panic attacks, defined as episodes of intense anxiety accompanied by symptoms of physical arousal. In this respect, consideration needs to be given to differentiate a real panic disorder from a panic attack precipitated by exercise in severe COPD patients, with a limited physical effort capacity. To be diagnosed with a pure panic disorder, patients must experience recurrent panic attacks, anticipatory anxiety about future attacks and some unexpected and unpredictable attacks (not always in situations inevitably causing dyspnea in COPD). The prevalence of panic disorder in COPD has been estimated as up to 10-times greater than the population prevalence of 1.5–3.5% [26, 27].

In our study, statistic outcomes revealed significant correlations of anxiety, depression and panic disorders with COPD symptoms intensity and with related quality of life, as assessed by mMRC and CAT. According to data published in 2011, by Salerno and Carone [28], there is a clear association between dyspnea and anxiety or depression in COPD, and these symptoms are correlated with and contribute to the severity of the chronic obstructive pulmonary disease. Authors concluded further studies are needed to better understand the relationship between psychological abnormalities and the physiopathology of COPD and how this may be further influenced by concurrent tobacco use.

Anxiety and depression are very often diagnosed in COPD, even in the same time, with a significant impact on patients’ quality of life and disease evolution. The reason for developing these symptoms is not very well known, but researchers have identified some frequently associated factors, like: low body mass index, physical disability, severe dyspnea, % of predicted FEV < 50%, co-morbidities, poor quality of life, female gender, current smoking, living alone and low social status [25]. Within the current study, all patients were smokers or ex-smokers and the scores for anxiety, depression and panic attacks were similar for both categories.

Studies from other countries show that even though health professionals are aware of the fact that symptoms of anxiety and depression are two of the most common co-morbidities in people with chronic obstructive pulmonary disease [29], in fact, it seems, these COPD co-morbidities are not routinely managed in all pulmonary diseases services, which leads to significantly poor health outcomes, reduced quality of life and increased healthcare costs [30].

Similar with other studies, our results recommend that clinicians caring for patients with COPD should screen for psychological distress and manage this co-morbidity appropriately [31]. According to current UK guidelines for the management of anxiety and depression, psychological treatment, pharmacological treatment or both in combination, are recommended as best practices [32, 33].

The limitations of the study are related to the relatively small sample size which was over represented by man participants, however this reflects the situation of the hospitalized patients with COPD. Moreover, the study did not include a control sample of people without COPD in order to compare the score for anxiety, depression and panic attacks between the groups, so the comparisons are possible only with data obtained by other studies.

## Conclusions

In conclusion, the results of this study indicate that anxiety, depression and panic attacks were constant characteristics among COPD patients, regardless of their current tobacco use. These findings underline the necessity to routinely screen anxiety, depression and panic in COPD patients. Further research is needed to assess the potential role of other tobacco related indexes with depression, anxiety, panick attacks among tobacco users with COPD.
